# Shoulder Rehabilitation Exercises With Kinematic Biofeedback After Arthroscopic Rotator Cuff Repair: Protocol for a New Integrated Rehabilitation Program

**DOI:** 10.2196/35757

**Published:** 2023-03-22

**Authors:** Ilaria Parel, Valeria Candoli, Maria Vittoria Filippi, Antonio Padolino, Giovanni Merolla, Stefano Sanniti, Riccardo Galassi, Paolo Paladini, Andrea Giovanni Cutti

**Affiliations:** 1 Laboratory of Biomechanics Azienda Unità Sanitaria Locale della Romagna Cattolica Italy; 2 Department of Clinical Engineering Azienda Unità Sanitaria Locale della Romagna Cesena Italy; 3 Department of Rehabilitation Medicine Azienda Unità Sanitaria Locale della Romagna Riccione Italy; 4 Shoulder and Elbow Surgical Unit Azienda Unità Sanitaria Locale della Romagna Cattolica Italy; 5 Laboratory of Motion Analysis National Institute for Insurance against Accidents at Work Prosthetic Center Vigorso di Budrio Italy

**Keywords:** rotator cuff tear repair, wearable sensor, biomechanic, eHealth, rehabilitation, scapula, biofeedback, kinematic, exercise, shoulder

## Abstract

**Background:**

The recovery of scapular and humeral physiological kinematic parameters, as well as the sensorimotor control of movement, plays a primary role in the rehabilitation process after arthroscopic rotator cuff repair. A highly customized rehabilitation approach is required to achieve this aim. Biofeedback can be a useful tool, but there is poor evidence of its application in the rehabilitation after arthroscopic rotator cuff tear repair.

**Objective:**

The aim of this paper is to outline an innovative exercise-based rehabilitation program exploiting visual biofeedback for the recovery of patients arthroscopically treated for rotator cuff repair.

**Methods:**

For establishment of the innovative program, a multidisciplinary team involving experts in shoulder surgery, rehabilitation medicine, physical therapy, and biomedical and clinical engineering was formed. Starting from a conventional rehabilitation program, the team selected a set of exercises to be integrated with a biofeedback tool, named the INAIL (National Institute for Insurance against Accidents at Work) Shoulder and Elbow Outpatient program (ISEO program). ISEO is a motion analysis system based on inertial wearable sensors positioned over the thorax, scapula, humerus, and forearm. ISEO can return a visual biofeedback of humerus and scapula angles over time or of the scapula-humeral coordination, with possible overlap of patient-specific or asymptomatic reference values. A set of 12 progressive exercises was defined, divided into four groups based on humerus and scapula movements. Each group comprises 2-4 of the 12 exercises with an increasing level of complexity. Exercises can require the use of a ball, stick, rubber band, and/or towel. For each exercise, we present the starting position of the patient, the modality of execution, and the target position, together with notes about the critical factors to observe. The type of visual biofeedback to adopt is specified, such as the coordination between angles or the variation of a single angle over time. To guide the therapist in customizing the patient’s rehabilitation program, a list of operative guidelines is provided.

**Results:**

We describe various applications of the ISEO exercise program in terms of frequency and intensity.

**Conclusions:**

An innovative rehabilitation program to restore scapular and humeral kinematics after rotator cuff repair based on kinematic biofeedback is presented. Biofeedback is expected to increase patient awareness and self-correction under therapist supervision. Randomized controlled trials are needed to investigate the potential effect of the exercise-based biofeedback in comparison with conventional rehabilitation programs.

**International Registered Report Identifier (IRRID):**

DERR1-10.2196/35757

## Introduction

Scapular dyskinesis is a common condition in a variety of shoulder disorders, including rotator cuff tears [1-5], with the medio-lateral and anterior-posterior scapular rotations most commonly affected [6]. The evaluation and treatment of scapular dyskinesis are challenging; in particular, finding accurate and repeatable measures to define “how much” scapular movement is dyskinetic and if it is a cause or effect of the rotator cuff tear is objectively difficult [7,8].

An exercise-based, manual, and proprioceptive treatment of scapular dyskinesis should be included in an individualized rehabilitation program following arthroscopic rotator cuff tear repair, with the purpose to restore pain-free physiological function of the shoulder [9]. Current rehabilitation programs should always refer to the time of tendon healing, surgical technique, evidence-based guidelines, literature, biomechanics, and kinesiology foundations, and should avoid a generic approach or a too-strict protocol [10-12]. However, the current literature lacks tools to specifically support therapists in the difficult challenge to tackle shoulder dyskinesis.

To address this aim, biofeedback systems might support patients in improving their own body function by receiving additional information about its status. This is typically achieved by sensing the target function though a wearable technology and returning this information to the patient through an alternative intrinsic sensory feedback (eg, audio, tactile, visual) [13].

Evidence about the use of biofeedback in rehabilitation of shoulder disorders remains limited in terms of both quantity and quality [14,15]. Some preliminary evidence suggests that biofeedback can improve scapular control in patients with adhesive capsulitis [16] and subacromial impingement syndrome, either based on electromyography (EMG) or simple video qualitative biofeedback [17-19]. However, there is poor evidence about the impacts of biofeedback based on quantitative kinematic data. In 2018, Hotta et al [20] reported a study on 26 subjects with impingement syndrome who were assessed during arm elevation before and after completion of a scapula training protocol based on kinematic biofeedback. No substantial differences were found for the range of motion (ROM) of the three scapular rotations, whereas the segmental coordination between the scapula and humerus showed significant differences with respect to scapular tilt in the lowering of the arm and with respect to internal rotation in the elevation and lowering of the arm. Opposite conclusions can be gathered from the study of Antunes et al [21], who completed a randomized controlled trial involving 30 healthy subjects divided into two groups. They tested the effectiveness of kinematic biofeedback on motor relearning transfer during shoulder flexion and a daily activity (drinking from a glass). All subjects were tested before and after the training. The training comprised scapular-focused exercises, performed with a real-time kinematic biofeedback tool consisting of 2D visual information of the scapulo-thoracic orientation. The results demonstrated that real-time kinematic biofeedback introduced relevant differences on scapula control and performance. These studies [20,21] refer to the immediate effect of a single-session biofeedback protocol based on few exercises. However, to our knowledge, there are no studies about the application of kinematic biofeedback for the recovery from rotator cuff tear surgical repair.

To fill this gap, we here propose a newly developed innovative exercise-based program, named the INAIL (National Institute for Insurance against Accidents at Work) Shoulder and Elbow Outpatient program (ISEO program), for the recovery of patients arthroscopically treated for rotator cuff repair. This program integrates the conventional rehabilitation program developed at the Service of Functional Recovery and Rehabilitation of the Azienda Unità Sanitaria Locale (AUSL) Romagna (Ceccarini Hospital, Riccione, Italy), with use of a kinematic visual biofeedback tool that provides a real-time objective and accurate evaluation of the upper arm kinematics [22].

## Methods

### Conventional Rehabilitation Program

The conventional rehabilitation program is based on a biomechanical foundation, the literature, clinical experience, time of tendon healing, and surgical instructions [10,23-25], providing therapists a timeline scheme with restrictions and objectives for each phase.

In phase 1 (day 1 to day 21), immobilization is established with use of an antirotation sling for 21 days. The sling can be removed for personal care or for active mobilization of the elbow, hand, and only sliding movements in elevation/depression and protraction/retraction of the scapula, keeping the gleno-humeral joint in resting position. Patients are instructed about how to wear and correctly remove the sling and about movement restrictions. Individual outpatient rehabilitation sessions start from day 15 and occur twice per week.

In phase 2 (day 21 to day 35), passive gleno-humeral mobilization on the scapular plane under a 90° angle is allowed (no rotational movements), as well as mobilization of the scapula. The sling is gradually removed from day 21 and passive ROM can be gradually increased over 90°. In this phase, basic exercises to activate scapular stabilizers and humeral head depressors are recommended, as well as improving postural alignment. It is crucial to avoid overstress of the repaired tendon. Two individual outpatient sessions per week are performed.

In phase 3 (day 35 to day 60), the aim is to restore full passive ROM, achieve good control of the kinematics, and correct the timing of muscles activation. From day 35, the patient can start performing gleno-humeral extra rotations and active humerus elevation exercises. Restoration of full passive ROM continues. In this phase, water exercises are integrated in the recovery program [26]. Four sessions per week are performed: two individual outpatient sessions and two sessions of aquatic therapy in a small group.

The final phase, phase 4 (from day 60 until return to work), occurs 2 months after surgery, in which dynamic strengthening exercises gradually advance using weights and rubber bands, with an increasing load on the repaired tendon. Functional exercises are integrated in the recovery program. In this phase, two individual outpatient sessions and, if still needed, two aquatic sessions per week are performed.

### ISEO Program

#### Overview

The ISEO program integrates use of instrumental kinematic biofeedback in the conventional rehabilitation program, focusing on the improvement of scapular control of patients recovering from arthroscopic rotator cuff tear repair. The ISEO program is based on recent literature about rotator cuff diseases [27,28], EMG and kinematic studies [29-34], shoulder biomechanical foundations [35], exercises described in other randomized controlled trials without biofeedback [36-40], and clinical experience. The ISEO program was developed following Consensus on Exercise Reporting Template (CERT) guidelines [41].

#### Device

The ISEO program is based on the previously described ISEO protocol application [22]. ISEO was validated against an optoelectronic system [22], and the agreement with respect to the “Scapula-Tracker” method was tested [42]. The reliability of ISEO was assessed, investigating the agreement both within and between testers [43], and age-dependent prediction bands of asymptomatic subjects were estimated [44]. To collect kinematic data, ISEO exploits an inertial and magnetic measurement system (Xsens Technologies, NL). This system is portable, small, easy to use, and comprises four wireless sensors that are placed over the thorax, scapula, humerus, and forearm.

Owing to a dedicated sensor-to-segment calibration and biomechanical model, ISEO provides a quantitative real-time evaluation of humerus and scapula rotations over time with respect to the thorax, as well as the scapulo-humeral coordination by measuring the three scapular rotations with respect to humerus elevation. Specifically, the scapulo-thoracic joint is described by means of three rotations: protraction-retraction, medio-lateral rotation, and posterior-anterior tilting [45]. The humerus orientation is described in terms of elevation with respect to the thorax in the sagittal (flexion-extension) and scapular (abduction-adduction) planes and in the axial rotation (external rotation [ER]/internal rotation [IR]). The scapular plane is intended to be 30°-40° anterior to the frontal plane.

**Figure 1:**
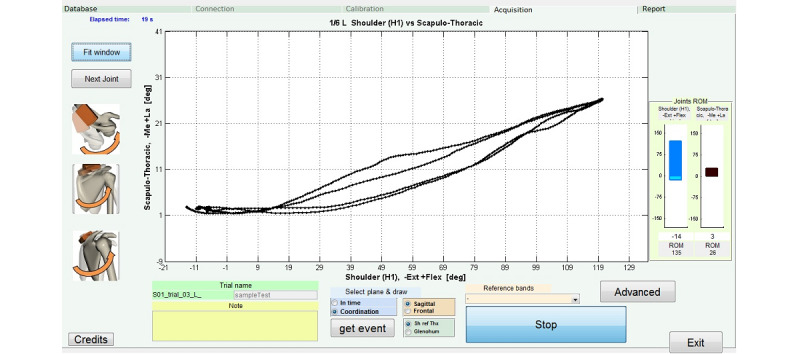
Scapulo-humeral coordination plot. Example of an angle-angle plot representing humeral flexion on the X-axis and scapula medio-lateral rotation on the Y-axis. L: left; H1: humerus [22]; Me-La: Medio-Lateral rotation; deg: degree; ROM: Range of Motion; Ext: extension; Flex: flexion; Sh ref Thx: shoulder referred to the thorax [22].

The system provides two types of real-time kinematic data plots, which are reported on a computer screen visible to both the therapist and the patient. The first is the scapulo-humeral coordination plot (Figure 1), which is an angle-angle plot representing humeral elevation on the X-axis (flexion-extension or abduction-adduction) and scapula rotations on the Y-axis (one plot for each scapula rotation). The patient can see how the scapula is moving while performing humerus flexion-extension or abduction-adduction. Preset reference bands [44], based on values obtained from asymptomatic subjects, can be used as targets during the exercises. Furthermore, a custom reference target can be created: the patient performs a reference movement with the help of the therapist, which can serve as the target for all subsequent exercises.

**Figure 2:**
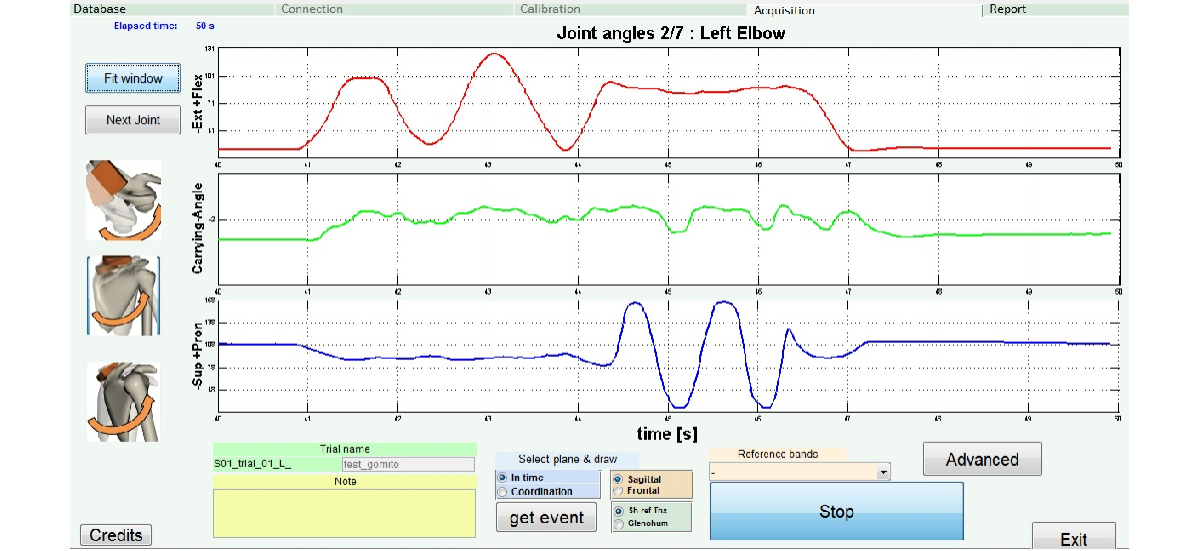
Time plot. Example of elbow kinematic data plotted with respect to time. Sup: supination; Pron: pronation.

The second is the time plot in which kinematic data are plotted with respect to time (Figure 2), representing humerus-thoracic and scapulo-thoracic rotations changes over time. The relative ROM is also updated in real time.

### Biofeedback Program Development

A multidisciplinary team was established for development of the ISEO program with the professionals typically involved in postoperative rehabilitation, patient assessment, and technology management. Specifically, the team comprised three experts in shoulder surgery and specific experience in shoulder rotator cuff arthroscopy (687 cumulative cases per year), two in rehabilitation medicine, two in physical therapy, and three in biomedical and clinical engineering (two with a PhD in shoulder biomechanics), each with more than 15 years of experience in the field.

After learning the ISEO motion analysis protocol, the team selected exercises from the conventional rehabilitation program as a starting point for integrating the biofeedback technology. In selecting and adapting the exercises, the team assumed the following constraints: (1) ISEO sensors shall not interfere with exercise execution and vice versa (eg, avoiding supine position or sensor contact with any physiotherapy equipment); (2) the therapist must not touch ISEO sensors during a possible manual interaction with the patient (eg, touching the shoulder); (3) the use of aids such as a ball, stick, and elastic band shall be preserved; (4) the visual biofeedback shall be clearly defined to facilitate the patient’s engagement and therapist interpretation; (5) exercises shall be performed while watching the visual interface on a screen in a comfortable position for both the patient and the therapist; (6) every exercise shall be monitored and directed by manual and/or verbal inputs by the therapist [46]; (7) to facilitate instructions to the patients, exercises shall only consider humerus movements in one of the main motion planes (sagittal, frontal, and transverse); and (8) exercises shall span all levels of skills, from early to end-phase rehabilitation.

Specifically, the multidisciplinary team selected 12 exercises. The two physical therapists and the two engineers then met during four 3-hour sessions to practice with the ISEO systems while performing the exercises. The two physical therapists, in turns, served as the test subject and therapist. This allowed them to have direct experience in exploiting the system from both the clinician and the patient perspective. Each exercise was checked in terms of respecting the selection constraints. During the practical sessions, engineers supported the physical therapists in identifying the best visual biofeedback to exploit, following the principles reported in the next section. At the end of the four practical sessions, the multidisciplinary team reconvened for a conclusive internal consensus meeting, to (1) review and confirm that all constraints were met, (2) group exercises based on the plane of moment, (3) define their progression, (4) define the physical therapy equipment, and (5) agree on the modality of visual biofeedback. Moreover, the modality of the intervention was set, together with a set of guidelines for therapists.

### Biofeedback Graphical Interface and Interpretation

The choice of the graphical interface to be used during the execution of a specific exercise is determined by the objective of the exercise itself.

If the main objective of an exercise is to work on a single joint angle (eg, ER/IR of the humerus with the scapula in a fixed position or scapular protraction/retraction with the humerus in a fixed position), a time plot of the angle of interest should be used. In this case, the main objective of an exercise is usually to obtain variation of the ROM (ie, ROM maximization for physiologic movements or ROM minimization for compensatory movements). The patient and therapist can monitor the trend and the ROM of the specific angle in real time.

If the main objective of an exercise is to work on a multisegmental coordination (eg, scapula medial rotation vs humerus sagittal elevation), then a scapulo-humeral plot should be used. This type of graph is particularly relevant for modulation of the scapulo-humeral coordination, which is usually altered in dyskinetic shoulders. The therapist can ask the patient to minimize or maximize the specific scapula movement during elevation of the humerus. Reference bands [44] can be used as the target (if indicated), and both the patient and the therapist can monitor the performance of the coordination in real time.

It should be considered that use of a time plot graph during an assessment and correction of a coordinated movement can lead to substantial loss of information, and therefore to a partial and potentially misleading feedback. By contrast, the use of a scapulo-humeral plot graph during a single joint movement can unnecessarily complicate the biofeedback graph and may not clearly highlight the kinematic trend of interest.

Figures 1 and 2 are examples of the graphs provided to both the therapist and the patient during execution of the exercises. During the first session, the therapist should instruct the patient with an extensive description of the graphs, and then a brief refresh session should be provided at the beginning of each exercise. Even if the patient is not capable of appreciating the biomechanical details of the graphs, they will be able to associate their movement variations to the curves plotted, and, with therapist assistance, understand the association between segmental movements and graph variations.

### Exercises

#### Overview

The ISEO program includes four groups of exercises (Table 1). The grouping is based on the plane of movement of the humerus (Groups 1 to 3) or of the scapula (Group 4). Each group includes a set of exercises, with a progression in difficulty and/or in load.

**Table 1:** List of the exercises included in the ISEO program.^a^

Exercise^b^			Physiotherapy equipment		Biofeedback
**Group 1**					
	A: Active assisted flexion/extension with a Bobath ball	Bobath ball		Coordination plots for the humerus elevation in the sagittal plane; time plot for ER/IR^c^ of the humerus with respect to the thorax	
	B: Active assisted flexion/extension with a stick	Stick		Coordination plots for the humerus elevation in the sagittal plane; time plot for ER/IR of the humerus with respect to the thorax	
	C: Active flexion/extension by stretching a rubber band	Rubber band		Coordination plots for the humerus elevation in the sagittal plane; time plot for ER/IR of the humerus with respect to the thorax	
	D: Active flexion/extension open chain	Optional: weight and/or rubber band		Coordination plots for the humerus elevation in the sagittal plane; time plot for ER/IR of the humerus with respect to the thorax	
**Group 2**					
	A: Active assisted abduction/adduction with a Bobath ball	Bobath ball		Coordination plots for the humerus elevation in the scapular plane; time plot for ER/IR of the humerus with respect to the thorax	
	B: Active abduction/adduction by stretching a rubber band	Rubber band		Coordination plots for the humerus elevation in the scapular plane; time plot for ER/IR of the humerus with respect to the thorax	
	C: Active abduction/adduction open chain without/with load	Optional: weight and/or rubber band		Coordination plots for the humerus elevation in the scapular plane; time plot for ER/IR of the humerus with respect to the thorax	
**Group 3**					
	A: Active ER/IR arm in adduction	Soft ball, towel, or pillow		Time plots for the three scapula rotations; time plot for ER/IR of the humerus with respect to the thorax	
	B: Active ER/IR at 90° flexion	Optional: weight and/or rubber band		Time plots for the three scapula rotations; time plot for ER/IR of the humerus with respect to the thorax	
	C: Active ER/IR at 90° abduction	Optional: weight and/or rubber band		Time plots for the three scapula rotations; time plot for ER/IR of the humerus with respect to the thorax	
**Group 4**					
	A: Push-up	None		Time plots for the three scapula rotations	
	B: Protraction-retraction at 90° and 120° of shoulder flexion	Optional: weight		Time plots for the three scapula rotations	

^a^ INAIL (National Institute for Insurance against Accidents at Work) Shoulder and Elbow Outpatient program.

^b^Grouping is based on the plane of movement of the humerus or of the scapula: Group 1=sagittal plane; Group 2=scapular plane; Group 3=external-internal rotation; Group 4=scapulo-thoracic.

^c^ER/IR: external rotation/internal rotation.

#### Group 1: Sagittal Plane

This group comprises four exercises characterized by movements of the humerus in the sagittal plane. The principal aim of the motor tasks is to achieve good scapular kinematic control while performing flexion and return. Coordination plots for the humerus elevation in the sagittal plane are used as visual biofeedback for monitoring the three scapula rotations.

The secondary aim is to check the associated ER of the humerus during elevation. For this purpose, the therapist selects the time plot as visual biofeedback to show the patient the active ROM in the ER/IR of the humerus with respect to the thorax.

The focus is on dynamic scapula stabilizer, infraspinatus, teres minor, and core stability (Textbox 1).

In this group, to ease trunk symmetry and stability, and avoid mistakes in scapulo-thoracic movement due to lateral tilt and/or rotation of the spine, all exercises are performed bilaterally, except exercise D that can be performed with one arm only. The progression from exercise C and D can be switched according to patient skills.

**Description of the exercises in Group 1 (sagittal plane).**

- **Exercise A: Active assisted flexion/extension with a Bobath ball**

**Figure 3:**
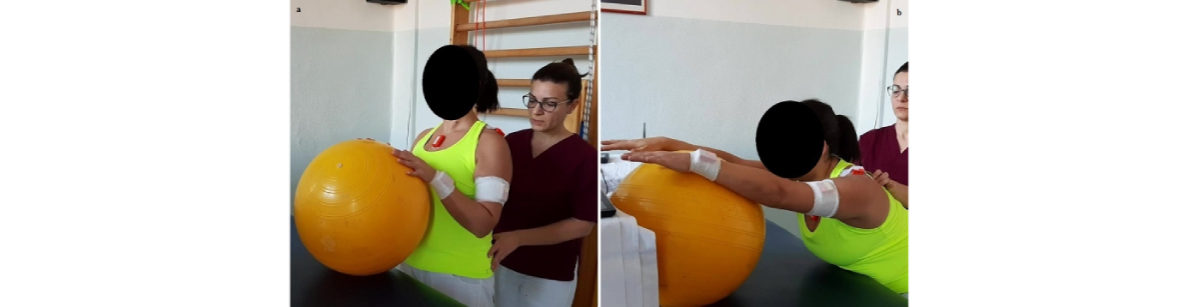
Group 1 (sagittal plane), exercise A: active assisted flexion/extension with a Bobath ball. (a) Initial position; (b) arrival position at maximum humerus flexion.

*Starting position:* standing with the Bobath ball on a bench, both hands over the ball at shoulder level, and the elbow flexed (Figure 3a).

*Execution*: to push the ball ahead using both arms.

*Target position*: standing position, humerus 90° flexed or more, elbows extended (Figure 3b).

*Notes*: as soon as the patient gains a wider gleno-humeral range of motion (ROM), they add flexion of the hips and lower back, with a stabilized trunk, to reach a humeral elevation of 120° or more. This exercise allows unloading the supraspinatus and deltoid. For this reason, it is the first to be performed.

- **Exercise B: Active assisted flexion/extension with a stick**

**Figure 4:**
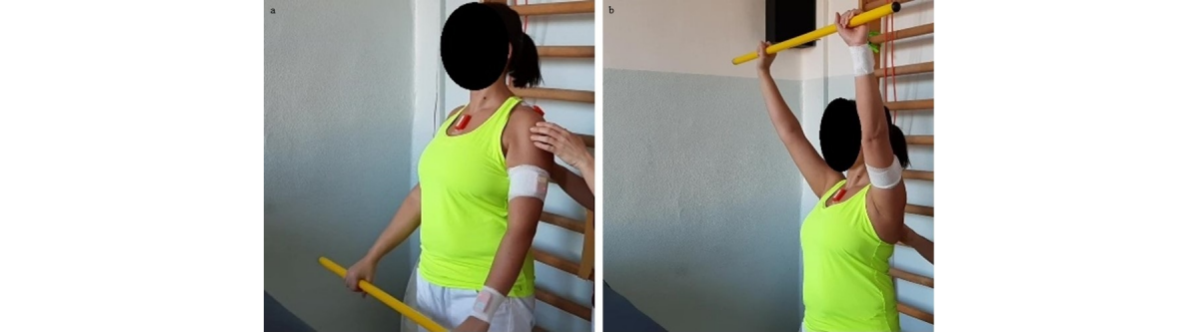
Group 1 (sagittal plane), exercise B: active assisted flexion/extension with a stick. (a) Initial position; (b) arrival position at maximum humerus flexion.

*Starting position*: standing or sitting position, hands hold the stick (the distance between the hands corresponds to the distance between the shoulders) (Figure 4a).

*Execution:* with the help of the other arm, the patient performs humerus elevation with the elbow extended.

*Target position*: standing or sitting position, humerus at 90° of flexion or more, elbows extended (Figure 4b).

*Notes*: it is important to check the trunk alignment and avoid hyperextension of the spine. If this happens, the exercise must be performed in sitting position and/or the target ROM must be reduced. If pain or inability to perform effective correction of scapular pattern occurs, the exercise can be performed with more distance between the hands, more assistance by the other arm, and/or changing the starting position of the hands (supinated, pronated, neutral).

- **Exercise C: Active flexion/extension by stretching a rubber band**

**Figure 5:**
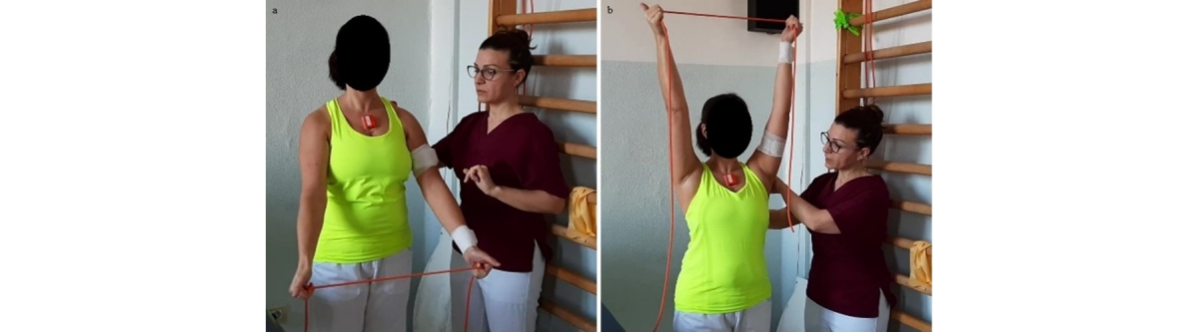
Group 1 (sagittal plane), exercise C: active flexion/extension by stretching a rubber band. (a) Initial position; (b) arrival position at maximum humerus flexion.

*Starting position*: standing or sitting position, elbow extended or at 90° of flexion, with a rubber band slightly stretched by both hands (Figure 5a).

*Execution*: the patient performs a small external rotation (reaching neutral position, with thumbs up) by stretching the rubber band, lifting up the arms with extended elbows while keeping the rubber band stretched and the humerus extra-rotated.

*Target position*: standing or sitting position, humerus at 90° flexion or more, elbows extended (Figure 5b). *Notes*: it is important to check trunk alignment and avoid hyperextension of the spine. The use of a rubber band provides greater activation of the infraspinatus and teres minor in comparison to the exercise performed without resistance.

- **Exercise D: Active flexion/extension open chain without/with load**

**Figure 6:**
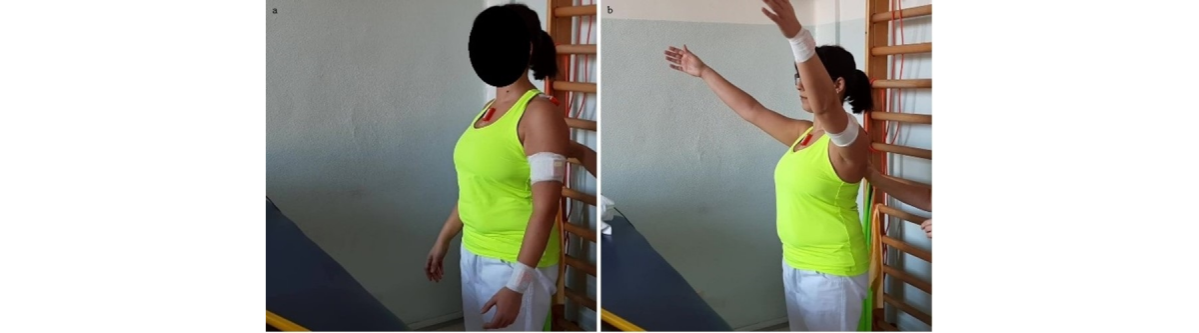
Group 1 (sagittal plane), exercise D: active flexion/elevation open chain without/with load. (a) Initial position; (b) arrival position at maximum humerus flexion.

*Starting position*: standing or sitting position, extended elbow (Figure 6a).

*Execution*: the patient lifts up the arms keeping both elbows extended.

*Target position*: standing or sitting position, humerus at 90° flexion or more, elbows extended (Figure 6b).

*Notes*: it is important to check the trunk alignment and avoid hyperextension of the spine; if performing with one arm, lateral flexion or rotation of the spine should be avoided. The exercise can be performed starting with 90° elbow flexion. Starting without a load is recommended as a first step; a weight or a rubber band can be progressively added later.

#### Group 2: Scapular Plane

This group comprises three exercises characterized by movements of the humerus in the scapular plane. The principal aim of the motor tasks is to achieve good scapular kinematic control during gleno-humeral abduction/adduction. Coordination plots in the scapular plane are used as visual biofeedback for monitoring the three scapula rotations during humeral elevation. The secondary aim is to check the associated ER of the humerus during elevation. For this purpose, the therapist selects the time plot as visual biofeedback to show the patient the active ROM in the ER/IR of the humerus with respect to the thorax.

The focus is on dynamic scapula stabilizer, infraspinatus, teres minor, and core stability (Textbox 2).

It is important to note that elevation on the sagittal and scapular plane is a functional motion (eg, reaching), and therefore needs to be trained as soon as possible and with accuracy. There is high activation of the serratus anterior and lower trapezius, resulting in lateral rotation of the scapula [32]. The lower trapezius maintains the instantaneous center of rotation of the scapula and helps the serratus anterior maintain the scapula close to the thorax, even if each muscle inserted in the scapula contributes to dynamic stabilization [3].

**Description of the exercises in Group 2 (scapular plane).**

- **Exercise A: Active assisted abduction/adduction with a Bobath ball**

**Figure 7:**
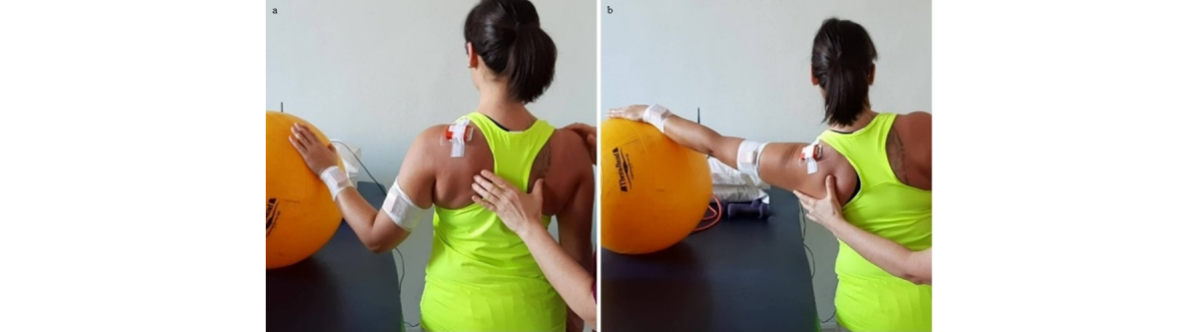
Group 2 (scapular plane), exercise A: active assisted abduction/adduction with a Bobath ball. (a) Initial position; (b) arrival position at maximum humerus abduction.

*Starting position*: standing position, Bobath ball on a bench or on the physiotherapy bed, hand over the ball at shoulder level, elbow in flexion (Figure 7a).

*Execution*: the patient pushes the ball sideways on the scapular plane.

*Target position*: standing position, shoulders at 90° flexion, elbow extended (Figure 7b).

*Notes*: as soon as the patient gains a wider gleno-humeral range of motion (ROM), they add low back lateral flexion to reach a humerus abduction of 120° or more.

- **Exercise B: Active abduction/adduction by stretching a rubber band**

**Figure 8:**
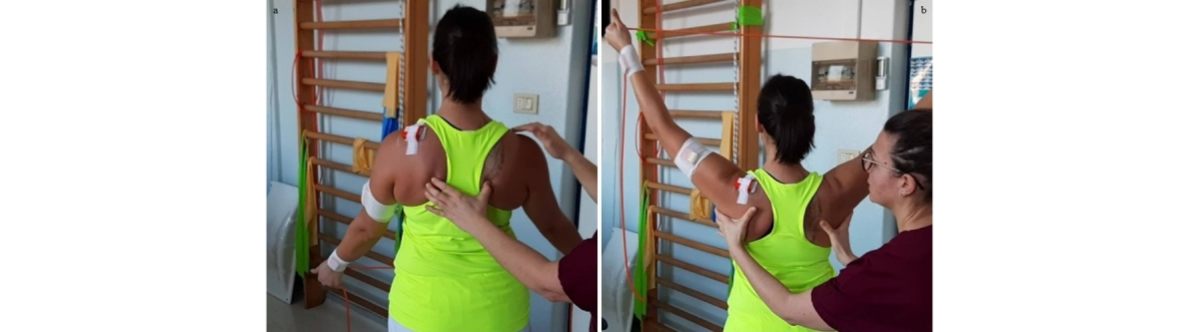
Group 2 (scapular plane), exercise B: active abduction/adduction by stretching a rubber band. (a) Initial position; (b) arrival position at maximum humerus abduction.

*Starting position*: standing or sitting position with extended elbows and a rubber band slightly stretched by both hands (Figure 8a).

*Execution*: the patient performs a small external rotation by stretching the rubber band, keeping both thumbs up, and then lifts up the arms on the scapular plane while keeping the rubber band stretched, the elbows extended, and the thumbs up.

*Target position*: standing or sitting position, humerus at 90° abduction on the scapular plane or more, elbows extended (Figure 8b).

*Notes*: it is important to check the trunk alignment and avoid hyperextension of the spine. The use of a rubber band provides greater activation of the infraspinatus and teres minor in comparison to the exercise performed without resistance.

- **Exercise C: Active abduction/adduction open chain without/with load**

**Figure 9:**
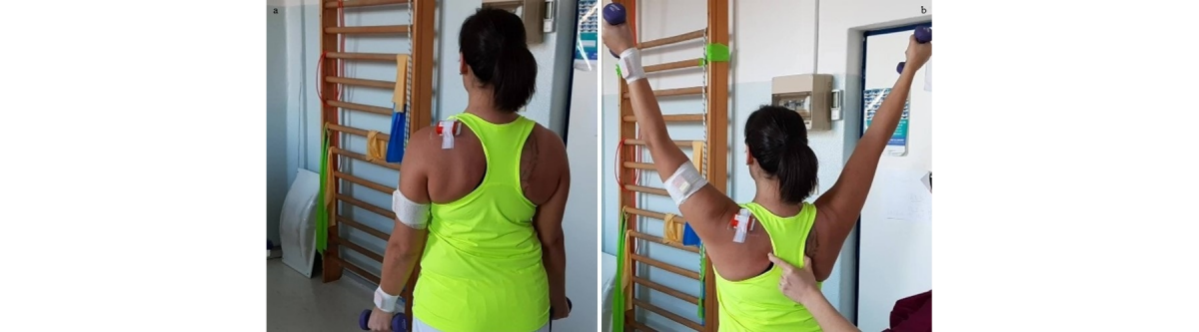
Group 2 (scapular plane), exercise C: active abduction/adduction open chain without/with load band. (a) Initial position; (b) arrival position at maximum humerus abduction.

*Starting position*: standing or sitting position with extended elbows (Figure 9a).

*Execution*: the patient lifts both arms upward.

*Target position*: standing or sitting position, humerus at 90° abduction on the scapular plane or more, elbows extended (Figure 9b).

*Notes*: it is important to check the trunk alignment and avoid hyperextension of the spine; if performing with one arm, the lateral flexion or rotation of the spine should be avoided. Starting without a load is recommended as the first step; a weight or a rubber band can be progressively introduced.

#### Group 3: External-Internal Rotation

This group comprises three exercises characterized by axial rotational movements of the humerus. The principal aim of this session is to achieve good scapular kinematic control during gleno-humeral ER and IR considering different humeral elevation positions. Time plots of the scapula are used as visual biofeedback to monitor the variation of the three scapula rotations during the execution of the exercise (eg, the patient must keep the line as flat as possible). The secondary aim is to achieve good active rotational ROM of the humerus and restore the strength of the rotator cuff muscles; time plots of the humerus with respect to the thorax are used as visual biofeedback for this purpose.

The focus is on dynamic scapula stabilizers, rotator cuff muscles, and core stability (Textbox 3).

Note that the progression for every exercise starts without a load, which can then be increased by using a rubber band or weights. Exercises B and C start with the arm laying on a horizontal support and progress without support (the patient keeps their arm lifted on their own). All exercises can be performed with both arms at the same time. The execution of the exercises in standing position requires higher core stability. In the case of poor postural core stability, the exercises can be performed with the back against the wall.

**Description of the exercises in Group 3 (external/internal rotation).**

- **Exercise A: Active external/internal rotation arm in adduction**

**Figure 10:**
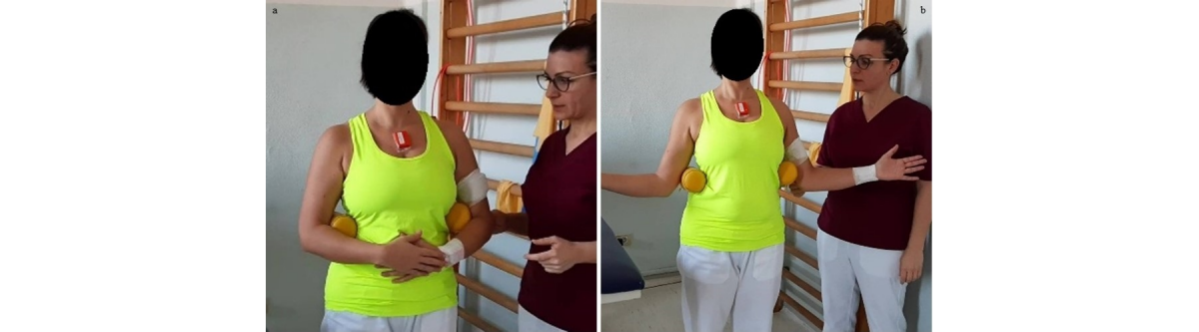
Group 3 (external-internal rotation), exercise A: active external/internal rotation arm in adduction. (a) Initial position; (b) arrival position at maximum humerus external rotation.

*Starting position*: standing or sitting position, arm adducted, hand on the belly, thumbs up, elbow 90° flexed, a small soft ball (or a towel or small pillow) between the elbow and trunk (Figure 10a).

*Execution*: the patient performs an external rotation of the humerus, keeping the ball fixed.

*Target position*: standing or sitting position, arm adducted, humerus externally rotated, elbow 90° flexed, thumbs up (Figure 10b).

- **Exercise B: Active external/internal rotation at 90° flexion**

**Figure 11:**
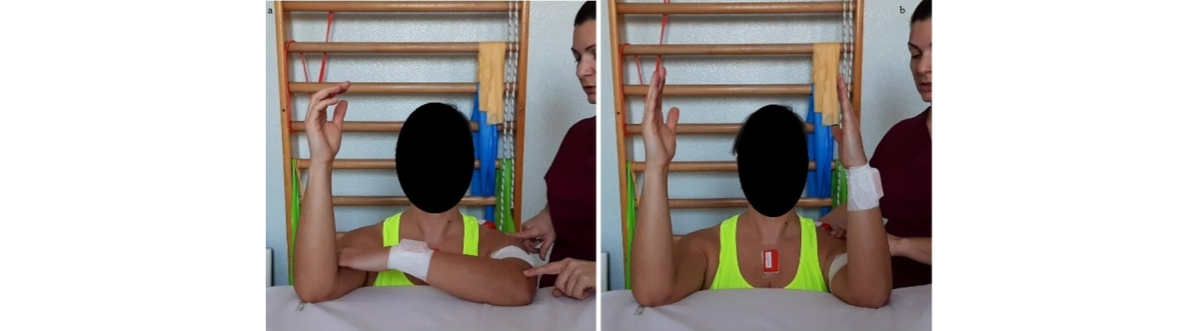
Group 3 (external-internal rotation), exercise B: active external/internal rotation at 90° flexion. (a) Initial position; (b) arrival position at maximum humerus external rotation.

*Starting position:* sitting or standing position, with the humerus at 90° flexion and internally rotated, elbow at 90° flexion, and forearm in horizontal position (Figure 11a).

*Execution*: the patient performs an external rotation of the humerus.

*Target position*: humerus at 90° flexion and externally rotated, elbow at 90° flexion, and forearm in vertical position (Figure 11b).

- **Exercise C: Active external/internal rotation at 90° abduction**

**Figure 12:**
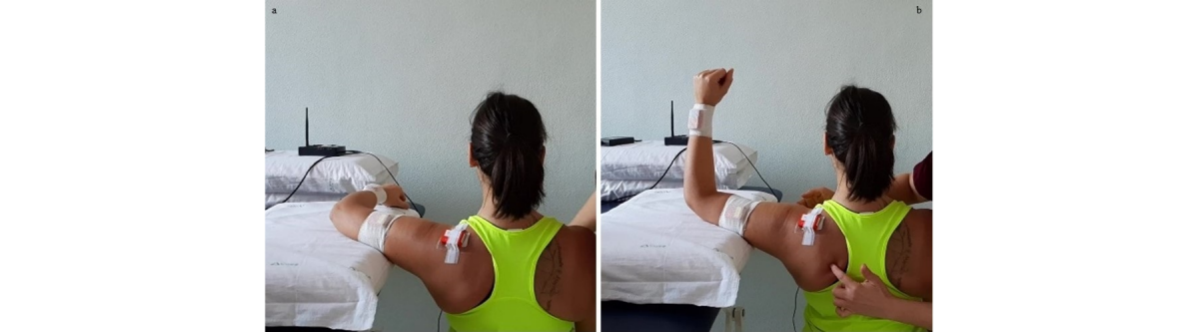
Group 3 (external-internal rotation), exercise C: active external/internal rotation at 90° abduction. (a) Initial position; (b) arrival position at maximum humerus external rotation.

*Starting position*: sitting or standing position, with the humerus at 90° abduction in the scapular plane and internally rotated, elbow at 90° flexion, and forearm in horizontal position (Figure 12a).

*Execution*: the patient performs an external rotation of the humerus.

*Target position*: humerus at 90° abduction and externally rotated, elbow at 90° flexion, and forearm in vertical position (Figure 12b).

#### Group 4: Scapulo-Thoracic

This group comprises two exercises characterized by movements of the scapula with respect to the thorax, while keeping the humerus in a predefined fixed elevation position. The principal aim of this session is to achieve strengthening of the serratus anterior together with the lower and medium trapezius and rhomboids. Scapula time plots are used as visual biofeedback to monitor the variation of the three scapula rotations during the execution of the exercises.

The focus is on the serratus anterior, trapezius, rhomboids, and core stability (Textbox 4).

**Description of the exercises in Group 4 (scapulo-thoracic).**

- **Exercise A: Push-up**

**Figure 13:**
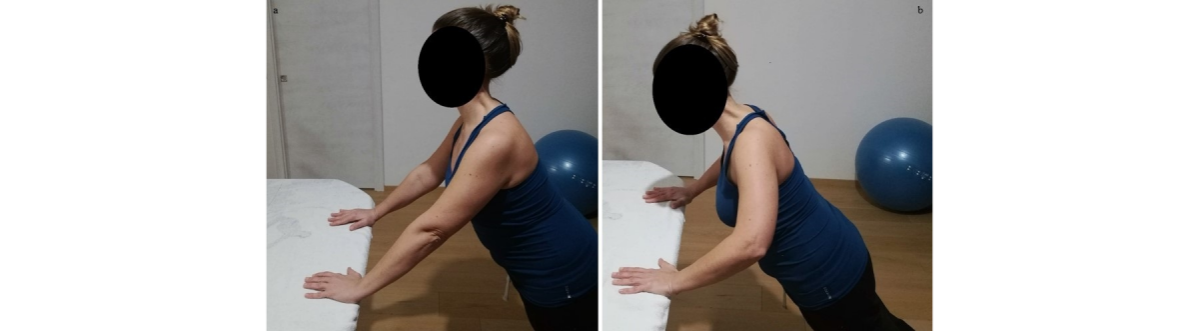
Group 4 (scapulo-thoracic), exercise A: push-up. (a) Initial position; (b) arrival position.

*Starting position*: feet on the ground, hands on a bench or on a physiotherapy bed (more height means less load), and humerus at 90° flexion (Figure 13a).

*Execution*: while keeping core stability, the patient performs elbow flexion/extension, nearing the body to the bench and back.

*Target position*: during the nearing, the elbows must stop before passing the line of the trunk (Figure 13b).

*Notes*: the therapist guides the patient to obtain good postural alignment and to stabilize the scapula. In this exercise, the scapula time plots have to be as flat as possible.

Weight-bearing exercises in a close kinetic chain show significantly greater muscle activation (mainly on the serratus anterior and infraspinatus) while increasing the load on the shoulder [29,34]. The progression of the exercise is from a standing, weight-bearing position to full weight-bearing (if possible).

- **Exercise B: Protraction-retraction at 90° and 120° shoulder flexion**

**Figure 14:**
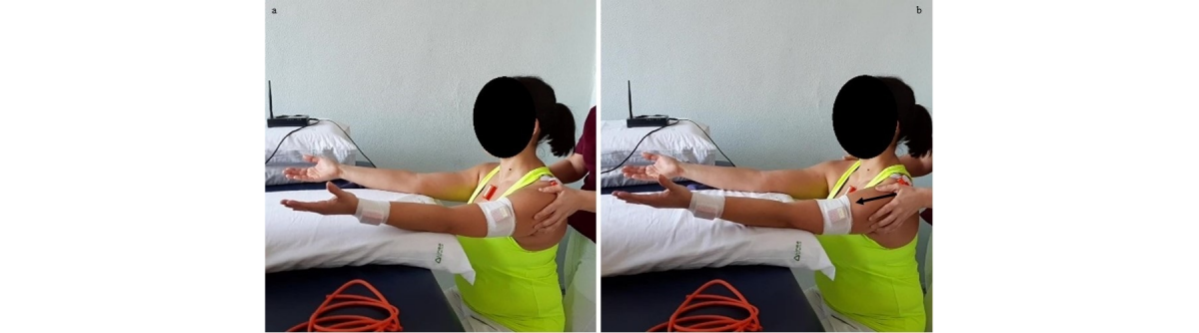
Group 4 (scapulo-thoracic), exercise B: protraction-retraction at 90° and 120° shoulder flexion. (a) Initial position; (b) arrival position.

*Starting position:* sitting position, humerus in flexion (between 90° and 120°), and elbows extended (Figure 14a).

*Execution*: while keeping core stability, the patient performs protraction and retraction of the shoulder girdle.

*Target position*: sitting position, humerus in flexion (between 90° and 120°), elbows extended, and shoulder girdle in maximum protraction (Figure 14b).

*Notes*: the exercise should be performed with both arms at the same time. In this exercise, the scapula time plots are used to check the variation of protraction-retraction and medio-lateral rotations. Progression starts with support of the elbow and forearm and then free (with or without holding a weight).

This exercise is specific for the serratus anterior and can be performed in standing position, but this requires higher core stability. In case of poor postural core stability, the exercises can be performed with the back against the wall.

### Guidelines for the Therapist

Textbox 5 provides operative guidelines to guide the therapist in customizing the patient's rehabilitation program.

**Operative guidelines for therapists during execution of the program.**

- **Assessment**

To choose the appropriate exercise and its starting level, the therapist first assesses the patient’s passive and active range of motion (ROM), the presence of pain at rest and during active movements, and the capability to maintain good postural control during active elevation.

- **Exercise presentation**

The therapist must provide a description and demonstration of the exercise to the patient, explaining what is shown on the screen and the visual goal to achieve.

- **Performance evaluation**

For every exercise, the aim is to achieve satisfactory neuromuscular control, without pain or compensatory strategies, with good postural alignment and core stability.

- **Intervention**

If an exercise is performed incorrectly and/or with compensation or pain, the therapist intervenes, giving physical and/or verbal guidance. The exercise can be modified (eg, decreasing the load, changing the starting position) or changed to an easier one.

- **Progression and load**

Any increase in difficulty and load must be gradual, individually adjusted, and with respect of the patient’s pain. Exercise progression can be achieved by increasing the ROM, the load, and changing the dosage and the starting position. All active exercises start with no weight other than the arm weight, and progress in 0.5-kg increments. Exercises with a rubber band (Rep band) start with light resistance (Level 1, yellow band) and progress with greater resistance (Level 2, orange band; Level 3, green band; Level 4, blue band). The progression of load occurs when the patient is able to perform a series of 10 repetitions for the specific exercise with no pain and any compensate. The assessment of scapula compensations can be performed using reference bands [44] provided for the scapulo-humeral coordination movements (while executing the specific movement, patient kinematic values should be within bands). If the time plot graph is used, the ROM of the specific angles should be observed and assessed: ROM maximization for physiologic movements and ROM minimization for compensatory movements.

## Results

We foresee the application of the exercise program twice a week, during individual physiotherapeutic sessions. Each session shall include 10 minutes for system preparation and calibration on the subject: sensors can be fixed over body segments (thorax, scapula, humerus, and forearm) by means of elastic bands and tape, following the previously detailed protocol [43]. The screen displaying the biofeedback graphs should be positioned to ensure a comfortable view for the patient and then 30 minutes of effective treatment will be conducted. Application of the ISEO program should start from the 45th day after surgery (or the timeline specified by surgeons) until the end of the rehabilitative treatment (ie, time of return to work).

## Discussion

### Strengths of the ISEO Program

We developed an innovative exercise-based program (ISEO program) for the recovery of patients arthroscopically treated for rotator cuff repair, which incorporates a kinematic visual biofeedback tool providing a real-time objective and accurate evaluation of the upper arm. We expect the biofeedback tool to support therapists and patients in the difficult challenge to tackle shoulder dyskinesis.

Specifically, with a multidisciplinary team of shoulder surgeons, physiatrists, physical therapists, and engineers, we defined a set of 12 progressive exercises divided in four groups based on the plane of movement of the humerus (Groups 1 to 3) or of the scapula (Group 4). Exercises can require the use of balls, sticks, rubber bands, and towels. For each exercise, the neuromotor goal and type of visual biofeedback is specified. Lastly, a detailed description of the method of administration of the protocol and a complete list of guidelines for therapists are provided for correct application and patient-specific customization (eg, administration of the progression and load for a single patient). To our knowledge, this is the first presentation of such a comprehensive rehabilitation program incorporating kinematics biofeedback; therefore, no direct comparisons to the literature are possible.

The ISEO rehabilitation program exploits quantitative motion analysis measures collected in real time by ISEO, owing to the use of wearable inertial and magnetic sensors. The ISEO protocol is extensively documented in the literature, with full details provided allowing its implementation on a wide range of possible hardware platforms. The documentation includes the availability of quantitative prediction bands for patient classification based on bootstrap statistical parameters. Reference bands were provided in Excel files as annexes of the main publication [44] for prompt availability. Commercial solutions are available by companies unrelated to the authors.

The description of the ISEO rehabilitation program is the first step toward randomized controlled trials investigating its potential efficacy and effectiveness in improving patients’ functional outcomes, activity level, and quality of life in comparison with conventional programs not incorporating kinematics biofeedback. In particular, it can be hypothesized that the ISEO program can be more effective than a conventional, nonvisual biofeedback protocol in increasing patient function in the short-term (90 days) and mid-term (1 year).

In this context, we believe that among many possible outcome measures, the following three would allow forming a global picture of the patients and might serve as primary outcome measures depending on the study aims.

### Outcome Measures

#### Functional Outcomes

The Scapula-Weighted Constant-Murley score [47], which is a modified version of the well-established Constant-Murley score for shoulder disorders, incorporating a specific assessment of scapula dyskinesis, was applied for the assessment of 32 patients arthroscopically treated for rotator cuff tear undergoing postoperative rehabilitation. The study showed that when scapula dyskinesis is accounted for, the number of patients with “good results” at 90 days postsurgery decreased from 31% to 9.5%, and at follow-up (after at least 6 months), the percentage dropped from 87.5% to 50%. This highlights the impact of scapula dyskinesis on the overall shoulder function and reinforces the common agreement that rehabilitation protocols should specifically address scapula dyskinesis [1,48].

#### Activities

The Disabilities of the Arm, Shoulder and Hand (DASH) score [49], exploring the impact of impairment on activities of the daily living, including work and recreational tasks, can be used as an outcome measure of activity. Psychometric properties specific for rotator cuff disease have been established supporting its use [50].

#### Health-Related Quality of Life

the EuroQuol-5D (EQ-5D) [51] instrument is an established measure of quality of life, which is recommended in cost economics and heath technology assessment studies, including interventions at the shoulder such as rotator cuff repair [52].

### Limitations

The ISEO program has limitations. A complete description of shoulder kinematics would also comprise sternoclavicular and acromioclavicular motion. Due to the size of the wearable sensors and the impossibility to reliably track segment position, the direct measure of sternoclavicular and acromioclavicular motion is not part of the current ISEO protocol. Therefore, their contribution to shoulder movement was not considered. The ISEO program does not have an age-range limitation, but requires patients to construct an intuitive understanding of the graphs and plots used for the visual feedback and link them to their own body motion. In the initial phase of the program, this requires some extra communication effort by the therapist to introduce the visual biofeedback to the patient. We might hypothesize that patients with a higher degree of confidence with digital systems can benefit the most or more quickly from the biofeedback system. In addition, the ISEO program was developed primarily for patients who underwent an anatomical rotator cuff repair and reconstruction. Finally, the ISEO program requires additional time for therapists to set up the system, and to troubleshoot hardware and software warnings, which can potentially slow down the active part of the therapy.

### Conclusions

We believe that the major strength of the ISEO program is providing a comprehensive tool that might effectively and quantitatively support rehabilitation professionals in tackling scapula dyskinesis, as recommended in the literature, by enhancing patient body awareness. Furthermore, the ISEO program can provide the opportunity to undertake a more engaging rehabilitation process, leveraging on the ludic aspect of the “digital feedback” and its novelty. The ISEO protocol provides quantitative parameters that can improve therapist-patient communication (as well as intra- and interprofessional communication), who can refer to objective enhancement or worsening of the kinematics over several sessions.

## Abbreviations

AUSL: Azienda Unità Sanitaria Locale
CERT: Consensus on Exercise Reporting Template
DASH: Disabilities of the Arm, Shoulder and Hand
EMG: electromyography
EQ-5D: EuroQuol-5D
ER: external rotation
INAIL: National Institute for Insurance against Accidents at Work
IR: internal rotation
ISEO: INAIL shoulder and elbow outpatient protocol
ROM: range of motion
